# Editorial: Atypical Development of Procedural Memory and Related Functions

**DOI:** 10.3389/fnhum.2022.957563

**Published:** 2022-06-22

**Authors:** Karolina Janacsek, Adam Takacs, Zsanett Tarnok

**Affiliations:** ^1^Institute of Psychology, ELTE Eötvös Loránd University, Budapest, Hungary; ^2^Brain, Memory and Language Research Group, Institute of Cognitive Neuroscience and Psychology, Research Centre for Natural Sciences, Budapest, Hungary; ^3^Centre of Thinking and Learning, Faculty of Education, Health and Human Sciences, School of Human Sciences, Institute for Lifecourse Development, University of Greenwich, London, United Kingdom; ^4^Cognitive Neurophysiology, Department of Child and Adolescent Psychiatry, Faculty of Medicine, TU Dresden, Dresden, Germany; ^5^Faculty of Medicine, University Neuropsychology Center, TU Dresden, Dresden, Germany; ^6^Vadaskert Child and Adolescent Psychiatry Hospital, Budapest, Hungary

**Keywords:** procedural memory, statistical learning, tourette disorder, dyslexia, developmental coordination disorder, developmental language disorder, retention

Learning skills and developing habits rely on procedural memory, a cognitive system rooted in the basal ganglia (Ullman, 2004; Janacsek et al., 2019, 2022). After repeated exposure to contingencies in the environment, long-lasting procedural memories emerge (Kóbor et al., 2017; Tóth-Fáber et al.). These memory representations contribute to automatic behavior from early in life (Janacsek et al., 2012; Juhasz et al., 2019). Recent studies on procedural memory and its interaction with other cognitive systems brought a new understanding of how people with developmental disorders learn new skills and create habits (Ullman and Pullman, 2015; Conway, 2020; Takacs et al., 2021). Curiously, atypical striatal development has been linked both to impaired and enhanced procedural memory functions. In the current issue of Frontiers in Human Neuroscience, we aimed to take a closer look into potential sources of the heterogeneity in this field.

Specifically, two review articles addressed the issue of procedural memory-related heterogeneity in dyslexia (Singh and Conway) and Tourette Disorder (TD) (Farkas et al.) separately. While these reviews are independent of each other, they show remarkable similarities that may explain heterogeneous results in these fields. For instance, they emphasized task heterogeneity and age-related variability effects. Importantly, they both highlighted the relevance of the multi-component (Singh and Conway) or multi-process (Farkas et al.) nature of procedural memory in atypical development. Furthermore, they both argued that not only learning but also retention is elemental to understanding the complexity between procedural systems and atypical development. The empirical studies of the current Research Topics all reflected the points raised by Singh and Conway and Farkas et al..

First, Lukács et al. studied the specific nature and timing of learning in developmental language disorder (DLD). While children with DLD had intact performance in the acquisition of acoustic verbal and visual non-verbal probabilities, they showed impairment in the metacognitive aspects of learning. The difference between learning indices and metacognitive scores sheds light on the distinction between online and offline measures of procedural learning and memory, especially in a developmental context. Namely, in the study of Lukács et al.. the pattern of online and offline indices suggested that online testing might be more sensitive and valid than offline tasks in atypical development. Moreover, it was suggested that the combined use of them would provide more clarity in cases when either learning or related metacognitive processes might be impaired.

Second, Tóth-Fáber et al. demonstrated the usefulness of the multi-component approach in TD: children and adolescents with TD learnt probabilistic information comparably to their typically developing peers. However, the TD group did not acquire serial order-based regularity. Thus, statistical and rule-based learning as different components of procedural memory contributes distinctively to the cognitive development of TD. Crucially, the relevance of these findings was highlighted by a one-year follow-up.

Third, Blais et al. presented a study on procedural learning in Developmental Coordination Disorder and or Developmental Dyslexia. They suggested that learning is modulated by the nature of cues which then leads to different retention patterns. Thus, procedural learning was not impaired in general terms in either of the groups. However, a process-oriented approach that considers how the information was presented might provide more optimal learning conditions in atypical development.

In sum, the studies on this Research Topic showed that the diverse development of procedural memory across modalities as well as its relationship to other cognitive systems could contribute to the heterogeneity of atypical cognitive functioning developmental disorders. Furthermore, this selection of theoretical and empirical works suggested future directions in concert, with a particular emphasis on neurophysiological research on the atypical development of procedural memory. We are hopeful that readers both from basic and clinical research and practice will find the presented studies useful and inspiring. We would like to thank the work of our authors and reviewers.

## Author Contributions

All authors listed have made a substantial, direct, and intellectual contribution to the work and approved it for publication.

## Funding

AT was supported by the Deutsche Forschungsgemeinschaft (DFG TA 1616/2-1).

## Conflict of Interest

The authors declare that the research was conducted in the absence of any commercial or financial relationships that could be construed as a potential conflict of interest.

## Publisher's Note

All claims expressed in this article are solely those of the authors and do not necessarily represent those of their affiliated organizations, or those of the publisher, the editors and the reviewers. Any product that may be evaluated in this article, or claim that may be made by its manufacturer, is not guaranteed or endorsed by the publisher.
